# Antimicrobial therapy outcomes in acute cholangitis: Hilar multiple obstructions *versus* single hilar and common bile duct obstructions

**DOI:** 10.1002/jgh3.13047

**Published:** 2024-03-14

**Authors:** Sakue Masuda, Yoshinori Imamura, Ryuhei Jinushi, Jun Kubota, Karen Kimura, Makomo Makazu, Ryo Sato, Makoto Kako, Masahiro Kobayashi, Haruki Uojima, Chikamasa Ichita, Kazuya Koizumi

**Affiliations:** ^1^ Department of Gastroenterology Medicine Center, Shonan Kamakura General Hospital Kamakura Kanagawa Japan; ^2^ Division of Medical Oncology/Hematology, Department of Medicine Kobe University Graduate School of Medicine Kobe Hyogo Japan; ^3^ Department of Gastroenterology Saitama Medical University International Medical Center Saitama Japan; ^4^ Department of Gastroenterology, Internal Medicine Kitasato University School of Medicine Sagamihara Kanagawa Japan

**Keywords:** acute cholangitis, antimicrobial resistance, antimicrobial stewardship, bile duct obstruction, hilar region

## Abstract

**Background and Aim:**

The appropriate duration of antimicrobial therapy for acute cholangitis (AC) arising from multiple hilar biliary obstructions as opposed to simple obstruction in the extrahepatic bile duct has not been established. This study assessed the efficacy of the duration of antimicrobial treatments in the Tokyo Guidelines 2018 for AC based on the cause and site of obstruction.

**Methods:**

This single‐center retrospective study involved patients with AC who underwent successful biliary drainage and completed a 7‐day or shorter antimicrobial treatment. Patients were categorized into three groups: Group 1, bile duct stone or benign obstruction; Group 2, simple biliary obstruction due to malignancy; and Group 3, multiple hilar biliary obstruction due to malignancy. The primary outcome was clinical cure rate, and the secondary outcomes were 3‐month recurrence rate and length of hospital stay.

**Results:**

A total of 373 patients were selected. Patients in Group 3 were younger or had Charlson Comorbidity Index ≥4, and had fewer positive blood cultures. In Group 3, the clinical cure rate (87.1%) and 3‐month recurrence rate (32.3%) were less favorable than those in the other groups. In Group 1, the clinical cure rate was significantly higher (98.1%, *P* = 0.02) with a much lower 3‐month recurrence rate of only 3.4% (*P* < 0.001) than that in the other groups. The median hospital stay for all groups was 7 days.

**Conclusion:**

This study suggests that the outcomes in Group 3 may be worse than those in Groups 1 or 2, regardless of the duration of the antibiotic treatment.

## Graphical abstract

This study evaluates the efficacy of antimicrobial therapy durations recommended by the Tokyo Guidelines 2018 for acute cholangitis (AC), comparing different obstruction etiologies. Results indicate that patients with multiple hilar biliary obstructions (Group 3) experience poorer outcomes compared to those with single obstructions (Groups 1 and 2), suggesting that treatment efficacy is influenced by obstruction complexity, irrespective of the antimicrobial therapy duration.

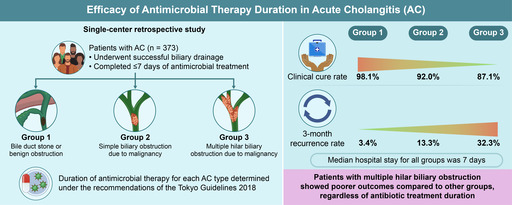

## Introduction

Acute cholangitis (AC), the second or fourth most prevalent cause of community‐acquired bacteremia,^1^, ^2^ is one of the most common diseases requiring antimicrobial therapy. The Tokyo Guidelines 2018 (TG18), which is among the most recognized AC guidelines, advocate a 4‐ to 7‐day antibiotic treatment duration following biliary drainage. However, robust evidence specifying the duration of antibiotic treatment to effectively treat AC is lacking.^3^ Recent research findings suggest that the brief use of antimicrobials, lasting only 1–3 days following biliary drainage, is comparable in efficacy to the conventional treatment duration for AC. This evidence proposes that administering antimicrobials for a short duration after biliary drainage represents a viable and reasonable approach for the treatment of AC.^4^, ^5^, ^6^

To our knowledge, no study has investigated whether AC resulting from multiple hilar biliary obstructions requires a similar duration of antimicrobial treatment as AC resulting from simple obstruction in the extrahepatic bile duct, which the TG18 guidelines treat equally.^3^ Bismuth‐IV obstruction (Figure S1, Supporting information) has been reported to have a higher clinical failure rate for bile duct drainage than other hilar obstruction.^7^ In patients with hilar biliary obstruction, the number of patients who required re‐drainage of the bile duct was higher in those treated with Bismuth‐II (91.9%), Bismuth‐IIIa (65.7%), and Bismuth‐IV (92.9%) obstruction than in those treated with Bismuth‐I (22.2%) obstruction.^8^ Therefore, the presence of multiple biliary obstructions may affect AC outcomes. It is unclear whether the standard treatment period recommended by the TG18 or the shorter treatment periods that have been reported to be effective for AC can be applied to cases of AC with multiple hilar biliary obstructions. Notably, performing cholangiography to determine the Bismuth classification may lead to bacteremia due to increased intrabiliary pressure.^9^ Therefore, depending on the severity of AC, sufficient cholangiography cannot be performed in some cases, making it difficult to accurately determine the Bismuth classification. In this study, we considered patients with multiple hilar biliary obstructions of Bismuth‐II or higher, which was determined from computed tomography and endoscopic retrograde cholangiopancreatography (ERCP) findings, as the group susceptible to poor drainage and adverse prognosis.

This study aimed to evaluate the efficacy of antimicrobial treatment lasting 7 days or less in cases of AC, as well as to investigate whether the clinical outcomes of cholangitis are affected by the presence of multiple hilar biliary obstructions or simple biliary obstructions in the extrahepatic bile duct.

## Methods

### Ethical considerations

This study was conducted in accordance with the 1964 Declaration of Helsinki and its subsequent amendments. This study was reviewed and approved by the Institutional Review Board of the Future Medical Research Center Ethics Committee (approval no. TGE01849‐024). Informed consent was obtained from all participants using the opt‐out method following the latest Japanese ethical guidelines.^10^ This approach was selected because the observational nature of the study relied on medical records, without involving samples derived from the human body.

### Study design and patient selection

This was a single‐center, retrospective cohort study. We included patients who presented with AC at Shonan Kamakura General Hospital between January 2018 and June 2020. AC was defined according to the TG18.^11^ Only patients aged ≥18 years who received a course of antibiotic treatment lasting within a 7‐day period following successful ERCP, in accordance with TG18 recommendations, were included. Technical success was defined as the placement of plastic or metallic stents above the bile duct obstruction or stones, or successful stone extraction.^7^

We excluded cases complicated by acute cholecystitis and those at the terminal stage of a malignant tumor. Terminal cases were defined as those where death due to cancer was confirmed within 30 days. Patients with a severe illness before AC onset, unsuccessful biliary drainage, biliary hemorrhage, a history of intestinal reconstruction methods other than Billroth I, and those for whom the mortality status could not be ascertained after 30 days were also excluded. Moreover, cases where antimicrobial therapy duration after biliary drainage exceeded 7 days, as well as recurrent cases with a history of AC within the past 3 months, were also excluded from the study. These specific cases were excluded as they significantly impact the outcomes of AC due to factors unrelated to its characteristics.

### Exposure

Patients were categorized into three groups: Group 1, bile duct stone or benign obstruction; Group 2, Bismuth‐I (simple biliary obstruction due to malignancy); and Group 3, Bismuth‐II or higher (hilar multiple biliary obstruction due to malignancy). Groups 1 and 2 were defined as the simple extrahepatic bile duct obstruction group.

In cases of Bismuth‐II or higher with hilar multiple obstructions, stent placement principally involved the insertion of two or three 7 Fr plastic stents into both lobes, depending on the extent of separation. This was performed to ensure as much drainage of the entire biliary tract as possible. However, in instances of Bismuth‐II where complete disconnection at the hilum was not evident, only a single stent was placed based on the attending physician's judgment. In cases of unsuccessful ERCP, endoscopic ultrasound‐guided drainage was performed.

The clinical success of ERCP in achieving biliary drainage is often defined as a decrease in bilirubin levels by more than 50% from the pretreatment value, measured 2 weeks after the procedure, according to the Tokyo Criteria 2014.^12^ However, patients with AC are often discharged within a week, and this definition does not allow early determination of drainage effectiveness. Therefore, in this study, clinical success following ERCP or alternative biliary drainage procedures was defined as achieving a 50% decrease or normalization of total bilirubin or alanine aminotransferase levels within 1 week of the procedure.

We initially adhered to the principle of a 4–5‐day duration following the TG18 guidelines. However, toward the latter part of the study period, accumulating evidence favored shortening the duration to 1–3 days for mild to moderate cases,^4^, ^5^, ^6^ and our hospital encouraged the shorter duration. Conversely, in severe cases or those with limited improvement in clinical symptoms and laboratory data, the duration was extended up to 7 days on a case‐by‐case basis.

### Variables and outcomes

The variables measured in the study population included age, comorbidities, cause of AC, severity of AC based on the TG18, National Early Warning Score (NEWS), ERCP findings, antimicrobial therapy, and blood or bile culture findings. Blood cultures were collected before antibiotic administration and bile cultures were collected immediately after ERCP. Subsequently, we assessed the severity of AC based on the TG18,^11^ factors previously reported to exacerbate outcomes in cholangitis (age,^13^ Charlson Comorbidity Index [CCI],^14^, ^15^ primary disease as a malignant tumor,^16^, ^17^ and time from consultation to ERCP^18^), antimicrobial therapy duration,^7^ and NEWS within 24 h before the termination of antimicrobial administration.^7^

Cholangitis is frequently associated with polymicrobial infections. Blood cultures exhibit low sensitivity and may fail to detect the causative organism, whereas bile cultures have low specificity and may detect enteric bacteria unrelated to the causative organism. This discrepancy complicates accurate identification of the causative organism of cholangitis. In this study, all identified bacteria were considered causative organisms. Interpretive standards from the Clinical and Laboratory Standards Institute for minimum inhibitory concentration or zone diameter testing were used to identify susceptible or resistant organisms.^19^

We considered antimicrobial therapy duration as an important factor to investigate based on previous reports indicating that it can be shortened compared with the recommended duration in the TG18.^4^, ^5^, ^6^

To assess the conditions of patients immediately before the discontinuation of antibiotics, we incorporated NEWS into our analysis. The United Kingdom's National Early Warning Score Development and Implementation Group developed the NEWS to assess deteriorating conditions in hospitalized patients and to predict inpatient death or intensive care unit (ICU) admission.^20^ NEWS measures physiological parameters (systolic blood pressure, pulse rate, respiratory rate, temperature, and oxygen saturation), consciousness level, and oxygen supplementation, all of which are simple and easily accessible.^20^, ^21^ The reports placed the low‐risk group based on NEWS at ≤4 points. NEWS is utilized in several countries due to its superior capability in identifying patients at risk for the composite outcome of cardiac arrest, unexpected ICU admission, and death within 24 h compared with other early warning scores. Furthermore, the Deyo modification of the CCI was used to define the severity of the comorbid conditions.^22^ A CCI score of ≥4 has been associated with poorer AC outcomes.^14^, ^15^, ^23^

The primary outcome was clinical cure, and the secondary outcomes were the 3‐month recurrence rate and length of hospital stay. Clinical cure was defined as the absence of initial presenting symptoms by day 14 after biliary drainage, coupled with no recurrence or death by day 30.^24^, ^25^ Recurrence was defined as the initiation of a new antibiotic therapy for recurrent cholangitis, subsequent infection in the hepatic–pancreatic–biliary region, or any other subsequent infection possibly related to the initial episode of cholangitis.^5^, ^26^, ^27^

### Statistical analysis

The three groups were classified based on the duration of antibiotic use after ERCP, and blood culture results were compared using univariate analysis. Continuous and categorical variables were reported as medians and interquartile ranges, and numbers and percentages, respectively. Continuous and categorical variables were compared using the Mann–Whitney *U* and chi‐square tests, respectively. Risk differences with 95% confidence intervals were calculated for binary outcomes, and a two‐sided significance level for all tests was set at *P* < 0.05. All analyses were performed using the EZR software version 1.55,^28^ a package for R statistical software (https://www.r-project.org/), which is a modified version of the R commander designed with statistical functions frequently used in biostatistics.

## Results

### Patient characteristics

**Figure 1:**
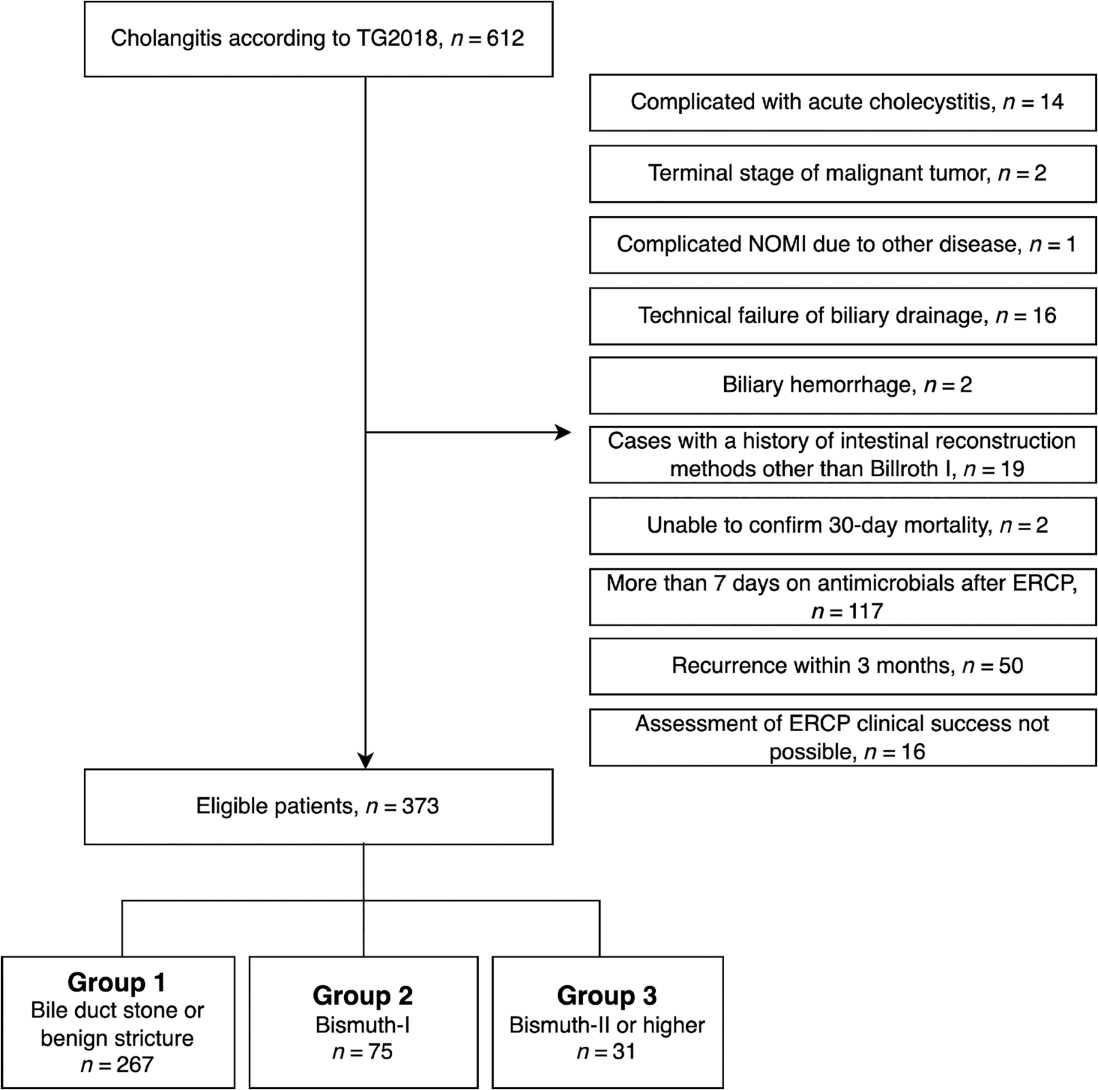
Flowchart of the inclusion and exclusion criteria. ERCP, endoscopic retrograde cholangiopancreatography; NOMI, non‐occlusive mesenteric ischemia; TG2018, Tokyo Guidelines 2018.

In total, 373 patients with AC were included in this study (Fig. 1). Table 1 summarizes the patient characteristics. Compared with Group 1, Group 3 comprised a greater proportion of younger patients or those with a CCI ≥ 4. Although there was no statistically significant difference, there were fewer cases of nursing home residents in Group 3. Numbers of severe cases or cases with high NEWS before the completion of antibiotic treatment were comparable between the Groups 1 and 3.

**Table 1:** Patient characteristics

	Group 1 benign	Group 2 Bismuth‐I	Group 3 Bismuth‐II or higher	
	*n* = 267	*n* = 75	*n* = 31	*P*‐value
Age, median [IQR]	81.00 [72.00, 87.00]	81.00 [70.00, 85.00]	73.00 [69.00, 83.00]	0.039
Age ≥75, *n* (%)	191 (71.5%)	50 (66.7%)	13 (41.9%)	0.004
Male, *n* (%)	130 (48.7%)	44 (58.7%)	13 (41.9%)	0.198
Cause of cholangitis, *n* (%)				
Bile duct stone	265 (99.3%)	0 (0.0%)	0 (0.0%)	
Malignant obstruction (distal bile duct cancer)	0 (0.0%)	23 (30.7%)	0 (0.0%)	
Malignant obstruction (pancreatic cancer)	0 (0.0%)	37 (49.3%)	0 (0.0%)	
Malignant obstruction (gallbladder cancer)	0 (0.0%)	3 (4.0%)	15 (48.4%)	
Malignant obstruction (hilar cholangiocarcinoma)	0 (0.0%)	6 (8.0%)	12 (38.7%)	
Malignant obstruction (cancer of other organs)	0 (0.0%)	6 (8.0%)	4 (12.9%)	
Benign obstruction and others	2 (0.7%)	0 (0.0%)	0 (0.0%)	
Severity of AC, *n* (%)				0.78
Mild	125 (46.8%)	36 (48.0%)	13 (41.9%)	
Moderate	112 (41.9%)	34 (45.3%)	15 (48.4%)	
Severe^†^	30 (11.2%)	5 (6.7%)	3 (9.7%)	
Underlying medical conditions, *n* (%)				
CCI (median [IQR])	1.00 [0.00, 1.00]	1.00 [0.00, 2.00]	1.00 [0.00, 4.00]	0.296
CCI ≥4	6 (2.2%)	7 (9.3%)	9 (29.0%)	<0.001
AIDS.HIV, *n* (%)	0 (0.0)	0 (0.0)	0 (0.0)	NA
Any malignancy including lymphoma and leukemia except malignant neoplasm of skin, *n* (%)	12 (4.5)	6 (8.0)	2 (6.5)	0.473
Cerebrovascular disease, *n* (%)	45 (16.9)	6 (8.0)	3 (9.7)	0.114
Chronic pulmonary disease, *n* (%)	6 (2.2)	2 (2.7)	1 (3.2)	0.933
Congestive heart failure, *n* (%)	12 (4.5)	2 (2.7)	0 (0.0)	0.395
Dementia, n (%)	24 (9.0)	5 (6.7)	1 (3.2)	0.475
Diabetes with chronic complications, *n* (%)	4 (1.5)	1 (1.3)	0 (0.0)	0.79
Diabetes without chronic complications, *n* (%)	84 (31.5)	24 (32.0)	13 (41.9)	0.497
Hemiplegia or paraplegia, *n* (%)	0 (0.0)	0 (0.0)	0 (0.0)	NA
Metastatic solid tumor, *n* (%)	0 (0.0)	6 (8.0)	6 (19.4)	<0.001
Mild liver disease, *n* (%)	9 (3.4)	0 (0.0)	3 (9.7)	0.036
Moderate or severe liver disease, *n* (%)	2 (0.7)	0 (0.0)	2 (6.5)	0.009
Myocardial infarction, *n* (%)	8 (3.0)	3 (4.0)	0 (0.0)	0.54
Peptic ulcer disease, *n* (%)	12 (4.5)	2 (2.7)	0 (0.0)	0.395
Peripheral vascular disease, *n* (%)	8 (3.0)	1 (1.3)	0 (0.0)	0.467
Renal disease, *n* (%)	6 (2.2)	1 (1.3)	0 (0.0)	0.634
Rheumatic disease, *n* (%)	4 (1.5)	0 (0.0)	0 (0.0)	0.448
Nursing home resident	61 (22.8%)	11 (14.7%)	3 (9.7%)	0.094
Immunosuppressant user	3 (1.1%)	2 (2.7%)	0 (0.0%)	0.469
Duration of antimicrobial therapy after biliary drainage, median [IQR]	4.00 [3.00, 5.00]	4.00 [2.00, 5.00]	3.00 [2.00, 4.50]	0.192
Duration of antimicrobial therapy 3 days or less, *n* (%)	97 (36.3%)	34 (45.3%)	16 (51.6%)	0.129
Highest NEWS within 24 h before the termination of antimicrobial administration (median [IQR])	1.00 [0.00, 2.00]	1.00 [0.00, 2.00]	1.00 [0.00, 1.00]	0.116
Highest NEWS of ≥5 within 24 h before the termination of antimicrobial administration, *n* (%)	5 (1.9%)	1 (1.4%)	0 (0.0%)	0.721

† During this trial period, there were no cases requiring intensive care unit admission among the 38 severe cases.

AC, acute cholangitis; CCI, Charlson Comorbidity Index; CHF, chronic heart failure; CKD, chronic kidney disease; DM, diabetes mellitus; ERCP, endoscopic retrograde cholangiopancreatography; IQR, interquartile range; LC, liver cirrhosis; NEWS, National Early Warning Score.

### ERCP findings

Table 2 outlines the ERCP findings. In Group 3, more patients had a longer time between the start of AC treatment and ERCP. However, when the time between the start of AC treatment and ERCP was limited to within 48 h, the proportion of patients in each group was comparable. The clinical success rates of biliary drainage and ERCP‐specific complications were also comparable between groups.

**Table 2:** ERCP findings

	Group 1 benign	Group 2 Bismuth‐I	Group 3 Bismuth‐II or higher	
	*n* = 267	*n* = 75	*n* = 31	*P*‐value
Time from consultation to ERCP, *n* (%)				0.053
≤24	208 (77.9%)	53 (70.7%)	17 (54.8%)	
24–48	36 (13.5%)	14 (18.7%)	10 (32.3%)	
>48	23 (8.6%)	8 (10.7%)	4 (12.9%)	
ERCP drainage procedure, *n* (%)				
Stone extraction	201 (75.3%)	1 (1.3%)	0 (0.0%)	
Plastic stent	52 (19.5%)	26 (34.7%)	28 (90.3%)	
Self‐expandable metallic stent	3 (1.1%)	38 (50.7%)	2 (6.5%)	
ENBD	4 (1.5%)	2 (2.7%)	1 (3.2%)	
Others	1 (0.4%)	0 (0.0%)	0 (0.0%)	
Stone extraction and stent placement/ENBD/others	4 (1.5%)	1 (1.3%)	0 (0.0%)	
Technical unsuccess of ERCP^†^	2 (0.7%)	7 (9.3%)	0 (0.0%)	
Clinical success of biliary drainage, *n* (%)	257 (96.3%)	72 (96.0%)	30 (96.8%)	0.982
Complications, *n* (%)				
Pancreatitis	5 (1.9%)	1 (1.3%)	0 (0.0%)	0.719
Bleeding	7 (2.6%)	1 (1.3%)	0 (0.0%)	0.548
Perforation	0 (0.0%)	0 (0.0%)	0 (0.0%)	NA
Others	0 (0.0%)	0 (0.0%)	0 (0.0%)	NA
No complications	253 (94.8%)	70 (94.6%)	31 (100.0%)	0.423

† All 9 cases of unsuccessful ERCP were transitioned to endoscopic ultrasound‐guided drainage.

ERCP, endoscopic retrograde cholangiopancreatography; IQR, interquartile range; ENBD, endoscopic nasobiliary drainage.

### Microbial culture findings

The results of the microbial cultures are summarized in Table 3, which shows a lower incidence of positive blood cultures in Group 3. Blood culture results were comparable between the groups.

**Table 3:** Blood and bile culture results

	Group 1 benign	Group 2 Bismuth‐I	Group 3 Bismuth‐II or higher	
	*n* = 267	*n* = 75	*n* = 31	*P*‐value
Blood culture, *n* (%)				<0.001
None taken	63 (23.6%)	44 (58.7%)	16 (51.6%)	
Negative rate	107 (40.1%)	17 (22.7%)	11 (35.5%)	
Positive rate	97 (36.3%)	14 (18.7%)	4 (12.9%)	
*Escherichia coli*	52 (19.5%)	5 (6.7%)	3 (9.7%)	0.017
*Klebsiella* sp.	22 (8.2%)	4 (5.3%)	0 (0.0%)	0.192
*Enterococcus* sp.	8 (3.0%)	1 (1.3%)	0 (0.0%)	0.467
*Enterobacter* sp.	7 (2.6%)	1 (1.3%)	0 (0.0%)	0.548
*Citrobacter* sp.	3 (1.1%)	1 (1.3%)	0 (0.0%)	0.823
*Streptococcus* sp.	5 (1.9%)	1 (1.3%)	0 (0.0%)	0.719
*Pseudomonas* sp.	1 (0.4%)	0 (0.0%)	0 (0.0%)	0.82
Anaerobes	10 (3.7%)	1 (1.3%)	2 (6.5%)	0.387
Others	8 (3.0%)	3 (4.0%)	1 (3.2%)	0.91
Bile culture, *n* (%)				<0.001
None taken	5 (1.9%)	5 (6.7%)	4 (12.9%)	
Negative rate	31 (11.6%)	26 (34.7%)	5 (16.1%)	
Positive rate	231 (86.5%)	44 (58.7%)	22 (71.0%)	
*Escherichia coli*	108 (40.4%)	18 (24.0%)	2 (6.5%)	<0.001
*Klebsiella* sp.	72 (27.0%)	18 (24.0%)	3 (9.7%)	0.106
*Enterococcus* sp.	98 (36.7%)	12 (16.0%)	12 (38.7%)	0.003
*Enterobacter* sp.	27 (10.1%)	8 (10.7%)	5 (16.1%)	0.591
*Citrobacter* sp.	17 (6.4%)	4 (5.3%)	4 (12.9%)	0.336
*Streptococcus* sp.	20 (7.5%)	8 (10.7%)	3 (9.7%)	0.651
*Pseudomonas* sp.	11 (4.1%)	1 (1.3%)	1 (3.2%)	0.507
Anaerobes	20 (7.5%)	3 (4.0%)	4 (12.9%)	0.262
Others	19 (7.1%)	5 (6.7%)	6 (19.4%)	0.053

Multiple bacteria were detected in bile cultures, which were all counted. In Group 3, the prevalence of *Escherichia coli* was lower, whereas *Enterococcus* spp. were more frequently observed.

### Antimicrobial therapy

Table 4 summarizes the antimicrobial therapy regimens in different groups. The most commonly used antibiotic was cefmetazole, followed by piperacillin/tazobactam and ampicillin/sulbactam. The broad‐spectrum antibiotic piperacillin/tazobactam was more commonly used in Group 3 than in the other groups. The duration of antibiotic administration after ERCP tended to be shorter in Group 3, although there were no significant differences. When considering all bacteria identified in the blood and bile cultures as causative organisms, the cases with resistance to the administered antibiotics were comparable between Groups 1 and 3. However, the bacteria identified in Group 2 showed less resistance.

**Table 4:** Antimicrobial therapy.

	Group 1 benign	Group 2 Bismuth‐I	Group 3 Bismuth‐II or higher	
	*n* = 267	*n* = 75	*n* = 31	*P*‐value
Cefmetazole, *n* (%)	166 (62.2%)	56 (74.7%)	15 (48.4%)	0.026
Piperacillin/tazobactam	35 (13.1%)	7 (9.3%)	8 (25.8%)	0.074
Ampicillin/sulbactam	43 (16.1%)	4 (5.3%)	2 (6.5%)	0.026
Ceftriaxone	12 (4.5%)	4 (5.3%)	2 (6.5%)	0.867
Meropenem	4 (1.5%)	1 (1.3%)	2 (6.5%)	0.146
Ciprofloxacin	4 (1.5%)	2 (2.7%)	1 (3.2%)	0.681
Vancomycin	0 (0.0%)	0 (0.0%)	0 (0.0%)	NA
Others	2 (0.7%)	1 (1.3%)	1 (3.2%)	0.435
Antimicrobial susceptibility for blood and bile cultures, *n* (%)				<0.001
Resistant bacteria	114 (42.7%)	18 (24.0%)	13 (41.9%)	
Susceptible bacteria	122 (45.7%)	29 (38.7%)	9 (29.0%)	
Unknown	31 (11.6%)	28 (37.3%)	9 (29.0%)	

IQR, interquartile range.

### Clinical outcomes

Table 5 presents the clinical outcomes of the patients. In Group 3, the clinical cure rate was 87.1%, and the 3‐month recurrence rate was 32.3%, which was less favorable than that in the other groups. In Group 2, the clinical cure rate was 92.0% and the 3‐month recurrence rate was 13.3%. In Group 1, the clinical cure rate was significantly higher at 98.1% (*P* = 0.002), with a much lower 3‐month recurrence rate of 3.4% (*P* < 0.001). The median hospital stay for all groups was 7 days.

**Table 5:** Clinical outcomes.

	Group 1 benign	Group 2 Bismuth‐I	Group 3 Bismuth‐II or higher	
	*n* = 267	*n* = 75	*n* = 31	*P*‐value
Clinical cure, *n* (%)	262 (98.1%)	69 (92.0%)	27 (87.1%)	0.002
3‐month recurrence rate, *n* (%)				<0.001
Recurrence	9 (3.4%)	10 (13.3%)	10 (32.3%)	
No recurrence	249 (93.3%)	62 (82.7%)	18 (58.1%)	
Unknown	9 (3.4%)	3 (4.0%)	3 (9.7%)	
Length of hospital stay, median [IQR]	7.00 [5.00, 8.00]	7.00 [6.00, 9.00]	7.00 [6.00, 10.00]	0.268

IQR, interquartile range.

The outcome difference between Groups 1 and 3 was particularly marked. The difference in clinical cure rates between Groups 1 and 3 was 11.0%, and the difference in 3‐month recurrence rates was 28.9%. Between Groups 2 and 3, the difference in clinical cure rates was 4.9%, and the difference in 3‐month recurrence rates was 19.0%. These results were similar in an analysis limited to cases treated with a 3‐day or shorter antimicrobial therapy duration (Table S1).

A minority of cases, totaling 15 (4.0%), did not achieve clinical cure. The characteristics of these cases are presented in Table S2.

## Discussion

We compared the outcomes between patients categorized into three groups. Patients in Group 3 tended to be younger and more likely to have a CCI of 4 or higher. Additionally, this group had fewer instances of positive blood cultures and fewer cases residing in nursing homes. There were no significant differences in the severity of AC, NEWS, duration of antibiotic therapy after ERCP, or technical and clinical success rates of ERCP. When all bacteria identified in the blood and bile cultures were considered causative organisms, the proportion of bacteria resistant to the administered antibiotics was comparable between Groups 1 and 3. Our findings indicated that Group 3 had significantly worse clinical outcomes than Group 1, with a lower rate of clinical cure and a higher rate of 3‐month recurrence. Additionally, compared with Group 2, Group 3 showed worse outcomes.

In cases of multiple obstructions near the hilar bile ducts, there is a higher clinical failure rate for bile duct drainage when there are more extensive obstructions.^7^, ^8^ Moreover, one randomized controlled trial showed that unilateral drainage is related to worse clinical success rates and an increased incidence of stent dysfunction compared with bilateral stenting in patients with hilar multiple biliary obstructions.^29^ However, to the best of our knowledge, there are no reports comparing the outcomes of AC with multiple bile duct obstructions with those of AC with simple bile duct obstruction.

Our investigation suggests that the outcomes of patients with AC with multiple hilar biliary obstructions may be poor. Although it is challenging to fully align our definition of outcomes with those of previous reports, the clinical cure rate of Group 3 was 87.1%, which is inferior when compared with those of Groups 1 and 2 or previous reports.^4^, ^5^, ^6^ Unlike the simple obstruction in the bile ducts outside the liver, in cases of multiple hilar biliary obstructions, it is sometimes difficult to achieve complete drainage of the entire bile duct. In cases of hilar multiple biliary obstructions, if drainage is achieved in more than 50% of the liver parenchyma's volume, there tends to be an improvement in blood test results, reflected in the bilirubin, alanine aminotransferase, or c‐reactive protein levels.^7^, ^30^, ^31^, ^32^ However, some bile duct branches may remain undrained. This study also achieved clinical success with bile duct drainage in most cases in all groups; however, the outcomes for Group 3 were poor. It should be noted that, even with the clinical success of biliary drainage, a complete cure for AC may not be achieved. We believe that the branches of the bile ducts that were not properly drained prevented full recovery from AC. This may also explain the high recurrence rate within 3 months.^8^, ^29^ Consequently, hilar multiple biliary obstructions may affect the outcomes of cholangitis. This suggests that proper treatment approaches may differ from simple obstructions of the bile ducts, particularly in the context of a standard or shorter duration of antimicrobial therapy.

Besides hilar multiple biliary obstructions, another factor that might have influenced the poorer outcomes seen in Group 3 was the CCI of ≥4. Multiple obstructions in the bile ducts can sometimes be caused by cancer metastasis from other organs. In our analysis of the CCI breakdown for Group 3, we found a notably higher prevalence of metastatic solid tumors and frequent instances of liver impairment. However, it is important to note that this observation in Group 3 may not solely stem from obstructive jaundice; substantive liver impairment induced by metastatic solid tumors could also play a significant role. Due to the inherent complexities of data extraction from the diagnosis procedure combination database, distinguishing between these causes proved challenging. While these factors may contribute to the higher number of cases with a CCI ≥4 in Group 3, future research should aim to address these confounding factors for a more accurate understanding.

These results should be interpreted with caution considering some limitations. This was a single‐center retrospective study, and the duration of antimicrobial therapy was determined by the physician in charge. Furthermore, the number of cases in Group 3 and the number of events without a clinical cure were small. Additionally, as this study was descriptive in nature, it did not adjust for confounding factors. Therefore, validation through prospective studies is necessary.

In conclusion, this study suggests that, in cases of AC with hilar multiple biliary obstructions, even when drainage is technically or clinically successful, the outcomes may be worse than those of simple extrahepatic bile duct obstructions with standard or shorter antibiotic treatment durations. Therefore, we suggest that proper treatment approaches for cases of AC with multiple hilar biliary obstructions differ from those of AC with simple obstructions in the extrahepatic bile duct.

## Supporting information


**Figure S1.** Bismuth classification. (A) Tumor is located at the common hepatic duct, but below the confluence of the right and left hepatic ducts. (B) Tumor involves the confluence of the right and left hepatic ducts but does not extend into secondary biliary radicals. (C) Tumor extends into the right (Type IIIa) or left (Type IIIb) hepatic duct. (D) Tumor involves both right and left hepatic ducts and/or has multifocal spread affecting secondary biliary radicals on both sides.
**Table S1.** Analysis limited to cases treated with shortened duration of antimicrobial therapy.
**Table S2.** Characteristics of patients who did not show clinical improvement.

## Data Availability

The datasets generated and analyzed in the current study are available from the corresponding author upon reasonable request.
