# Supplementary material for: Antimicrobial therapy outcomes in acute cholangitis: Hilar multiple obstructions versus single hilar and common bile duct obstructions
Source: JGH Open. 2024 Mar 14;8(3):e13047. doi: 10.1002/jgh3.13047 (PMC10938463; doi:10.1002/jgh3.13047)
Supplement: Supplementary file 1 — Figure S1. Bismuth classification. (A) Tumor is located at the common hepatic duct, but below the confluence of the right and left hepatic ducts. (B) Tumor involves the confluence of the right and left hepatic ducts but does not extend into secondary biliary radicals. (C) Tumor extends into the right (Type IIIa) or left (Type IIIb) hepatic duct. (D) Tumor involves both right and left hepatic ducts and/or has multifocal spread affecting secondary biliary radicals on both sides. Table S1. Analysis limited to cases treated with shortened duration of antimicrobial therapy. Table S2. Characteristics of patients who did not show clinical improvement. [file JGH3-8-e13047-s001.docx]

**Supplementary Material**

**Figure S1.** Bismuth classification.
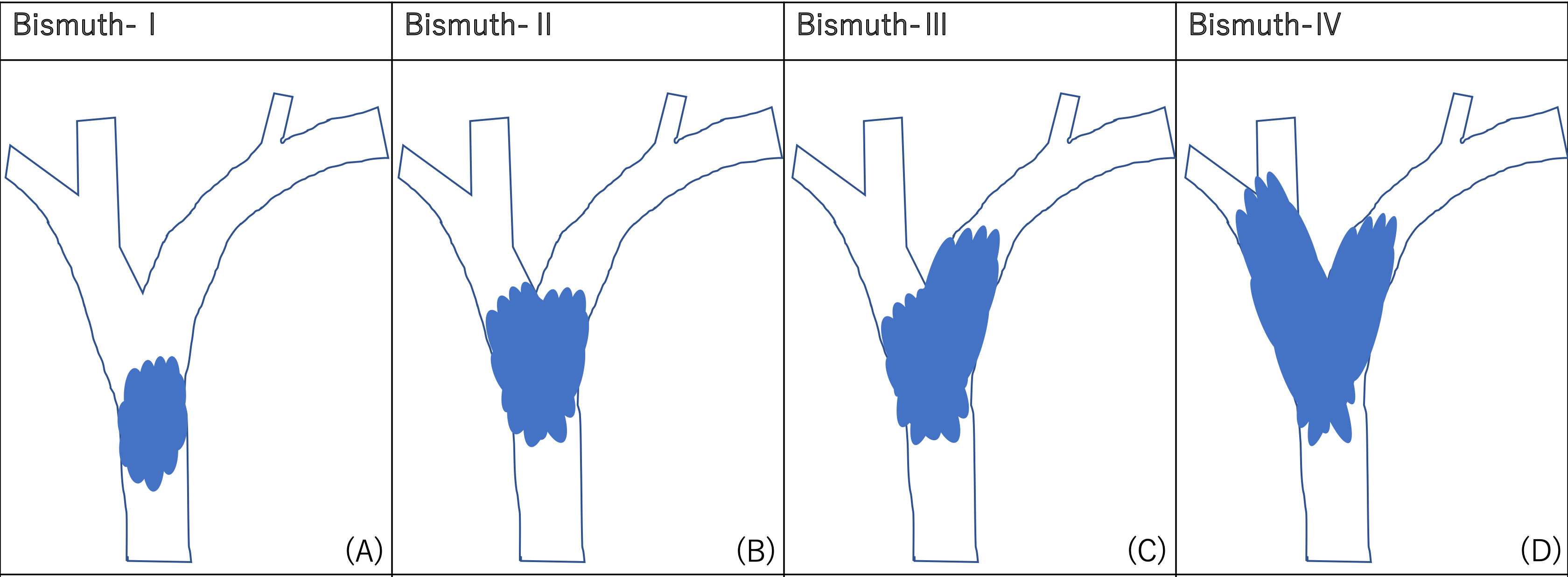


1. Tumor is located at the common hepatic duct, but below the confluence of the right and left hepatic ducts.
2. Tumor involves the confluence of the right and left hepatic ducts but does not extend into secondary biliary radicals.
3. Tumor extends into the right (Type IIIa) or left (Type IIIb) hepatic duct.
4. Tumor involves both right and left hepatic ducts and/or has multifocal spread affecting secondary biliary radicals on both sides.

**Table S1.** Analysis limited to cases treated with shortened duration of antimicrobial therapy.

|  | **Group 1** Benign | **Group 2** Bismuth-Ⅰ | **Group 3** Bismuth-Ⅱ or higher | p-value |
| --- | --- | --- | --- | --- |
|  | n=267 | n=75 | n=31 |  |
| Clinical cure, n (%) | 97 (100.0%) | 31 (91.2%) | 14 (87.5%) | 0.005 |
| 3-month recurrence, n (%) |  |  |  | 0.004 |
| Recurrence | 5 (5.2%) | 5 (14.7%) | 5 (31.2%) |  |
| No recurrence | 89 (91.8%) | 28 (82.4%) | 9 (56.2%) |  |
| Unknown | 3 (3.1%) | 1 (2.9%) | 2 (12.5%) |  |
| Length of hospital stay, median [IQR] | 6.00 [5.00, 7.00] | 7.00 [6.00, 8.75] | 7.00 [5.75, 10.00] | 0.049 |

IQR, interquartile range.

**Table S2.** Characteristics of patients who did not show clinical improvement.

|  | No clinical cure | Clinical cure |
| --- | --- | --- |
|  | n=15 | n=358 |
| Age, median [IQR] | 80.00 [74.50, 86.00] | 80.00 [71.00, 87.00] |
| Malignant obstruction, n (%) | 10 (66.7%) | 96 (26.8%) |
| Bismuth classification, n (%) |  |  |
| I | 6 (40.0%) | 69 (19.3%) |
| II or higher | 4 (26.7%) | 27 (7.5%) |
| Bile duct stone/benign obstruction/Others | 5 (33.3%) | 262 (73.2%) |
| Blood culture, n (%) |  |  |
| Negative | 2 (13.3%) | 133 (37.2%) |
| Positive | 5 (33.3%) | 110 (30.7%) |
| No collection | 8 (53.3%) | 115 (32.1%) |
| Severity of AC, n (%) |  |  |
| Mild | 7 (46.7%) | 167 (46.6%) |
| Moderate | 6 (40.0%) | 155 (43.3%) |
| Severe | 2 (13.3%) | 36 (10.1%) |
| CCI≥4, n (%) | 2 (13.3%) | 20 (5.6%) |
| Clinical success of biliary drainage, n (%) | 13 (86.7%) | 346 (96.6%) |
| Technical unsuccess of ERCP, n (%) | 15 (100.0%) | 355 (99.2%) |
| Time from consultation to endoscopic retrograde cholangiopancreatography, n (%) |  |  |
| ≤24 | 12 (80.0%) | 266 (74.3%) |
| 24 - 48 | 2 (13.3%) | 58 (16.2%) |
| >48 | 1 (6.7%) | 34 (9.5%) |
| Antimicrobial susceptibility for blood and bile cultures, n (%) |  |  |
| Resistant bacteria | 5 (33.3%) | 140 (39.1%) |
| Susceptible bacteria | 5 (33.3%) | 155 (43.3%) |
| Unknown | 5 (33.3%) | 63 (17.6%) |
| Short-course duration of antibiotics, n (%) | 5 (33.3%) | 142 (39.7%) |
| Highest NEWS of ≥5 within 24 h before the termination of antimicrobial administration, n (%) | 1 (7.1%) | 5 (1.4%) |

IQR, interquartile range; AC, acute cholangitis; CCI, Charlson Comorbidity Index; ERCP, endoscopic retrograde cholangiopancreatography; NEWS, National Early Warning Score.
